# Challenges and Advances in Diagnosis and Treatment of Leptomeningeal Disease (LMD)

**DOI:** 10.3389/fonc.2021.800053

**Published:** 2022-01-12

**Authors:** Sherise D. Ferguson, Elena I. Fomchenko, Renato A. Guerrieri, Isabella C. Glitza Oliva

**Affiliations:** ^1^ Department of Neurosurgery, University of Texas, MD Anderson Cancer Center, Houston, TX, United States; ^2^ Department of Melanoma Medical Oncology, University of Texas, MD Anderson Cancer Center, Houston, TX, United States

**Keywords:** leptomeningeal disease, ctDNA = circulating tumor DNA, targeted therapy, immunothearpy, intrathecal therapy

## Abstract

Leptomeningeal disease (LMD) is a devastating category of CNS metastasis with a very poor prognosis and limited treatment options. With maximal aggressive therapy, survival times remain short and, without treatment, prognosis is measured in weeks. Both LMD diagnosis and treatment are challenging topics within neuro-oncology. In this review, we discuss the advances in LMD diagnosis with a focus on the role of circulating tumor DNA (ctDNA) and discuss the role of targeted and immunotherapy in LMD treatment.

## Introduction

Leptomeningeal disease (LMD) is a dire category of central nervous system (CNS) metastasis that entails tumor cell dissemination to the cerebrospinal fluid (CSF) and/or leptomeninges and often results in significant neurological morbidity. It occurs in approximately 5-15% of patients with solid tumors, and the incidence is rising (1–8). Among solid malignancies, lung cancer, breast cancer and melanoma are most frequently associated with LMD. Further within each primary malignancy, specific subtypes have been linked with an increased propensity for LMD. In patients with non-small cell lung cancer (NSCLC), those harboring an epidermal growth factor receptor (EGFR) mutation have a higher risk of LMD; specifically 9.4% in EGFR-mutant tumors versus 1.7% in wild-type tumors in a retrospective analysis of patients with NSCLC LMD (9). Similarly in breast cancer, tumor subtype, specifically HER2 (human epidermal growth factor receptor 2) status has been described to impact LMD propensity and outcome (10, 11). For example, the triple negative molecular subtype (*estrogen/progesterone/HER2 negative*) accounts for approximately 40% of breast cancer LMD cases, however only is diagnosed in 10% of all breast cancer patients, indicating a clear overrepresentation of this subtype among LMD patients. Furthermore, the time from breast cancer diagnosis to the development of LMD is shorter in hormone receptor negative cases (12–14).

As mentioned, the incidence of LMD is rising across various tumor types, possibly due to the fact that patients live longer with both extracranial and intracranial parenchymal metastatic disease due to advances in systemic therapy and improved imaging. Autopsy reports suggest that the actual frequency of LMD may be underestimated. In fact, one postmortem analysis of patients with cancer who exhibited neurologic symptoms revealed that 18% had evidence of leptomeningeal infiltration (15). The spread of cancer cells can be local or disseminated through the entire CNS, and LMD almost always results in rapid neurologic disability and death. If untreated, survival can be as short at 4-6 weeks (5). However, even with currently available treatment options, prognosis remains dismal, and therefore, LMD represents one of the most challenging disease processes in neuro-oncology. In this review we will discuss advances in the diagnosis and treatment of LMD with a focus on targeted and immunotherapy.

## Challenges in LMD Diagnosis and the Role of ctDNA

**Figure 1:**
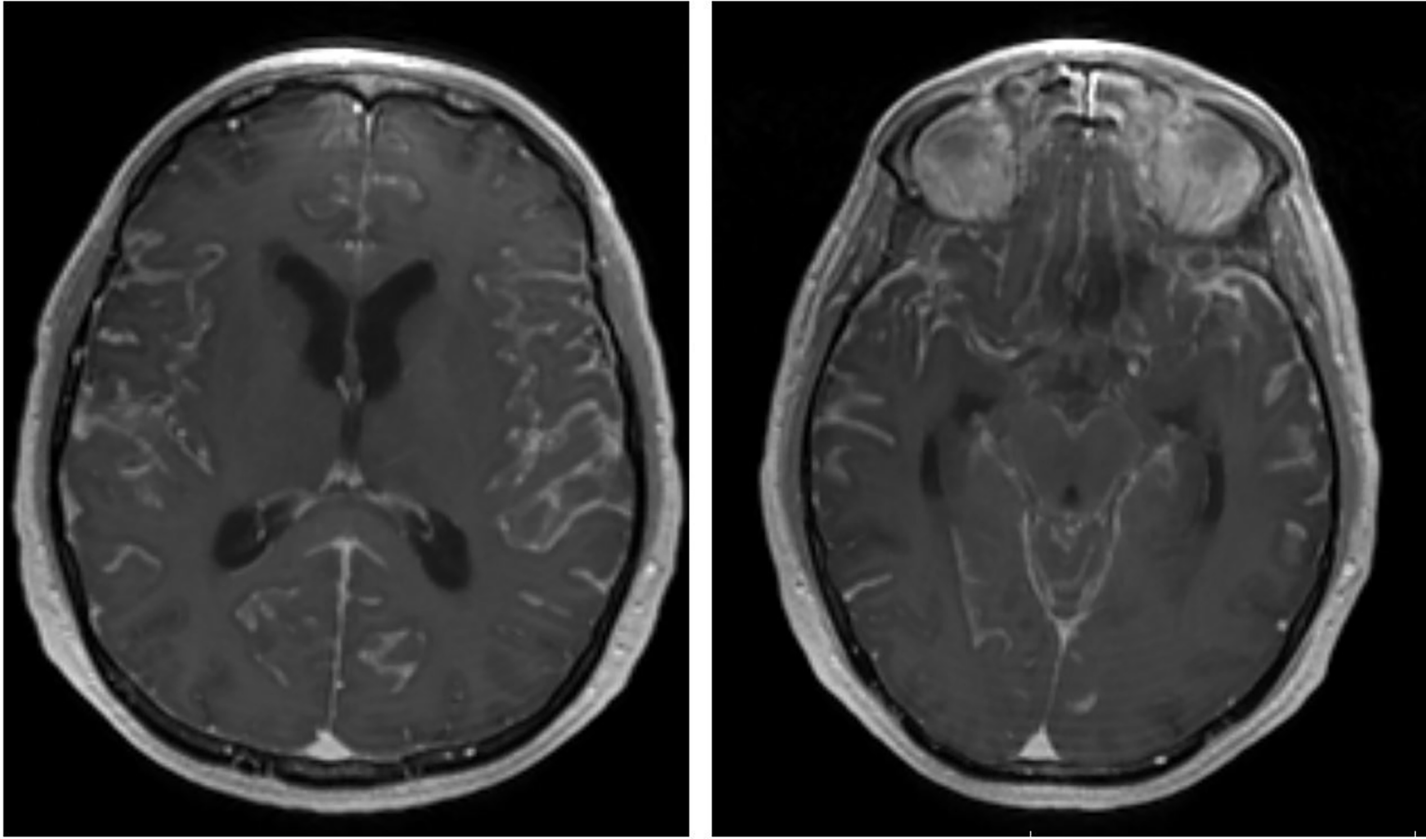
Representative post-contrast brain MRI (axial) showing diffuse LMD with enhancement within the cerebral sulci.

The diagnosis of LMD is currently based on neurological symptoms, contrast-enhanced magnetic resonance imaging (MRI) or computed tomography (CT) imaging characteristics and CSF cytopathology analysis. Since CSF tumor spread can propagate to the entire CNS, LMD can present with a wide constellation of neurological symptoms and signs from invasion of the brain, spine and/or cranial nerves. As such, symptoms may include (but not limited to) headache (from meningeal irritation), cranial nerve palsies, altered mental status, bowel or bladder dysfunction and/or extremity weakness. Furthermore, some patients may develop symptoms of elevated intracranial pressure from LMD induced aberrations in CSF dynamics. Radiographically, LMD may present with a variety of enhancement patterns on MRI. Classically, MRIs show linear or nodular enhancement along the cerebral sulci, dura, cerebellar folia, spinal cord/cauda equina and/or cranial nerves (16) (**Figure 1**). LMD disease can also cause abnormalities in CSF dynamics resulting in ventriculomegaly and potentially increased intracranial pressure. Finally, CSF analysis is critical in the diagnosis of LMD and is currently considered the gold standard. CSF is most commonly acquired *via* lumbar puncture and cytology may show the presence of malignant cells. In addition to cytology, CSF cell count and chemistry (particularly protein levels) are abnormal in 90% of LMD patients, aiding in diagnosis (17, 18).

Despite these approaches, the definitive diagnosis of LMD can still be quite challenging due to the limited sensitivity of initial CSF cytology and MRI and the high variability of presentation between patients. MRI has the benefit of being non-invasive, so it is typically the first diagnostic step; however, the sensitivity and specificity are 75% and 77%, respectively (19). Several clinical scenarios, including recent radiation or surgery, infection and intracranial hypotension, can resemble LMD by also causing abnormal enhancement. Furthermore, adjacent parenchyma metastasis can also cause localized sulcal/leptomeningeal enhancement. In situations where imaging is unclear or equivocal, CSF analysis is required; however, in many cases, CSF analysis is performed as an additional confirmatory step even when radiographic findings are consistent with LMD. Despite CSF analysis being the diagnostic gold standard, it still has significant limitations. First, CSF cytology has lower sensitivity than MRI, with malignant cells reportedly detected in only 50-67% of patients (17, 20–22). Frequently, two or three CSF samples are required to establish the diagnosis of LMD, as the sensitivity rises to 80-90% with a second and third lumbar puncture (17). In most cases, CSF chemistry, specifically elevated protein, can be observed, but these abnormalities are also non-specific. Finally, CSF cytological analysis may suffer from inter-observer variability and may depend on the experience of the pathologist (16).

Prompt diagnosis of LMD is critical, and improved diagnostics could potentially improve the outcome of these patients. Further, none of the currently available diagnostic modalities can provide reliable information on disease burden or (early) response to treatment. As such, novel molecular tools have emerged to address these issues. Considering the pathogenesis of LMD remains elusive, these tools also provide the opportunity to assess specific and potentially actionable therapeutic targets.

Among recent molecular advancements, circulating tumor DNA (ctDNA) has provided an avenue to address the above concerns. ctDNA are short, double stranded tumor DNA fragments that are released by tumor cells as a result of cell apoptosis and or necrosis. ctDNA are different from so called circulating tumor cells (CTCs), which are intact tumor cells (23, 24). The role of ctDNA has been demonstrated in multiple studies across different solid tumor types and includes aiding early diagnosis, monitoring treatment response and monitoring patients for early recurrence in the adjuvant setting (23, 25–27). Further, tumor-specific genomic abnormalities can be detected in ctDNA using next-generation sequencing techniques or PCR-based options, and this assay has demonstrated a strong concordance with the genomic profile of malignant tissue, making ctDNA a potentially powerful biomarker (23, 28).

In the setting of neurological malignancies, the utility of plasma-derived ctDNA is suboptimal (29–32). However, recent studies have shown that CSF is a promising platform for ctDNA analysis in the setting of intracranial pathology (31). For example, Mattos-Arruda et al., performed targeted sequencing and/or exome sequencing coupled with droplet digital PCR (ddPCR) on matched CSF-derived ctDNA, plasma-derived ctDNA, and tumor tissue deposits in patients with both primary and metastatic brain tumors (31). These authors reported significantly higher genomic alteration sensitivity in CSF-derived ctDNA than in plasma-derived ctDNA in patients with CNS tumors. Furthermore, this same study observed treatment-associated changes in CSF ctDNA signatures through longitudinal collection of CSF, potentially identifying new therapeutic targets. This finding has also been consistent in patients with LMD. In a study of patients with NSCLC LMD, Ying et al. performed sequencing on 72 matched CSF and plasma samples. Mutation detections rates were 81% in CSF versus only 62% in plasma. Moreover, the average maximum allelic frequency in plasma was 4.6% versus 44% in CSF, demonstrating that CSF is superior to plasma for mutation identification and genomic analysis of LMD (33).

In cases where CSF cytology and MRI finding are indeterminate, confirming the diagnosis of LMD is very difficult. Several studies have suggested that ctDNA can augment the diagnostic yield of CSF for prompt LMD diagnosis (34, 35). For example, Zhao et al. directly compared the sensitivity of imaging, CSF cytology and CSF ctDNA in the diagnosis of LMD. The authors evaluated CSF samples from 35 LMD cases across multiple primary malignancies (36). They reported that 71% of cases had positive CSF cytology and 63% had imaging features consistent with LMD while CSF ctDNA extraction and next-generation sequencing revealed cancer associated mutations in 100% of cases, highlighting the sensitivity of ctDNA. In a melanoma specific study, Ballester et al., utilized ddPCR and next-generation sequencing to evaluate the CSF-derived ctDNA of seven melanoma patients with LMD. First, this study confirmed the diagnostic power of ctDNA, as 30% of patients whose CSF samples were negative/indeterminate for malignant cells were positive for CSF ctDNA. Further, analysis of ctDNA showed the presence of melanoma associated mutations, and in two patients, they found a correlation between mutant allele fraction and radiographic tumor volume on MRI, suggesting that ctDNA could potentially aid in monitoring tumor burden (34). Since CSF can be collected serially at multiple time-points in the patients’ treatment course, small studies have investigated the role of ctDNA in assessing and monitoring LMD tumor burden pre- and post-treatment (36–39). In a cohort of patients with EGFR mutated, NSCLC LMD, Zheng et al., evaluated if ctDNA could assess response to EGFR targeted therapy with osimertinib (tyrosine kinase inhibitor). The authors reported that CSF ctDNA revealed the genomic landscape of LMD in patients prior to treatment with osimertinib and following progression on treatment. They reported that overall detection of EGFR-sensitizing mutations was greater than 93% in patients with EGFR-mutated NSCLC LMD. Further, detection of EGFR 19 deletion and positive T790M (a point mutation in the EGFR tyrosine kinase domain) in the CSF was associated higher median intracranial progression free survival on osimertinib. Concurrent cell cycle alterations with EGFR-sensitizing mutations were associated lower median intracranial progression free survival. Additionally, analysis of a cohort of patients whose CSF was genotyped following progression post osimertinib treatment revealed multiple potential resistance mechanisms (37). In summary, the utility of ctDNA has been shown in three main categories: first, to aid in early diagnosis; second, to assess treatment course and tumor burden; and third, to potentially uncover actionable targets to optimize or introduce novel treatment strategies.

## Role of Targeted Therapy in LMD

Historically, the treatment for LMD was divided into systemic therapy, radiation and intrathecal (IT) approaches. Until recently, systemic treatment options have been significantly limited due to the inability of chemotherapy to penetrate the blood brain barrier (BBB). However, recent advances in the treatment that may overcome this limitation, namely targeted therapy and checkpoint inhibitors, have brought some hope for patients with LMD. Moreover, while the vast majority of investigation in NSCLC, breast and melanoma is based on the systemically administered therapies, IT approaches are currently being investigated.

For NSCLC LMD, current therapies target EGFR mutations and anaplastic lymphoma kinase (ALK) rearrangement *via* tyrosine kinase inhibitors (TKIs). Targeting these molecular alterations has substantially impacted the prognosis of advanced NSCLC. Multiple studies have been published on systemic targeted therapy applications for patients with NSCLC LMD including case reports, retrospective and prospective studies, as well as phase 1 open label and phase 3 randomized controlled trials. In regards to EGFR inhibition, first generation and second generation TKIs such as gefitinib, erlotinib and afatinib were initially evaluated with some success but most have been challenged by limited CNS penetration (40–42). Magnification of therapy using higher concentrations or a pulsatile regimen has been proposed to increase the CSF concentrations (43–46). However, as a result of this limited BBB permeability, CNS recurrence following treatment with first generation and second generation TKIs is as high as 40% (47). Based on multiple studies, third-generation TKI osimertinib has shown notable promise in the treatment of EGFR mutated LMD due to its higher BBB penetration (48–53). Yang et al. published a phase I study of 32 patients with LMD patients treated with osimertinib in which 87% of patients reported symptomatic improvement and 72% had a radiographic response (48). The follow-up study of 41 patients reported functional improvement in 57% of patients, a response rate of 41% and overall survival (OS) of 11 months [The BLOOM study (49)]. In a recent phase II study evaluating osimertinib in patients with brain metastasis and LMD, the authors reported an intracranial control rate of 92.5% (complete response in 12.5%) and a median OS of 13 months (53). Overall, based on these data, osimertinib is considered a viable treatment option for EGFR mutated NSCLC. An ongoing multicenter phase 2 trial [Osimertinib in Patients With a Lung Cancer With Brain or Leptomeningeal Metastases With EGFR Mutation (ORBITAL)] will assess CNS response rates to osimertinib as a single agent (NCT04233021). Another phase 2 trial is evaluating the safety and efficacy of osimertinib in combination with bevacizumab (NCT04425681 and NCT04148898).

AZD3759 is an alternative novel EGFR inhibitor that is capable of efficiently crossing the BBB. The safety of AZD3759 in patients with EGFR-mutant NSCLC, brain metastases and LMD was evaluated in a multicenter phase I study (54). Incidentally, the authors observed significant radiographic efficacy in three of four enrolled LMD patients. Another phase I study by Cho et al. reported that AZD3759 showed a reduction in EGFR expression in the majority of cases and stable disease in 50% (55). Nimotuzumab, another new generation TKI, has undergone limited evaluation, but a cases series with promising results and rapid responses has been reported (56).

ALK mutations are rare and can be found in approximately 3–7% of patients with NSCLC; targeting this mutation has been a notable step in the treatment of this patient population (57). The data specifically addressing the role of ALK inhibition in LMD is less robust than addressing EGFR inhibition, however, some small studies have been supportive of this approach and have demonstrated efficacy in CNS disease. Frost et al. reported the recent result of a German early access program with lorlatinib, a third-generation ALK inhibitor. This study included 52 patients who had progressed on first and second-line ALK inhibitors. The majority of the included patients had CNS disease [nine (17%) had LMD specifically], and the authors reported an intracranial response rate of 54% (58). A recent phase 3 clinical trial compared lorlatinib with first generation ALK inhibitor crizotinib in 296 patients with advanced *ALK*-positive NSCLC who had received no previous systemic treatment for metastatic disease. Intracranial response was a secondary end point and a complete intracranial response was achieved in 71% of cases, highlighting that later generation ALK inhibitors have relatively higher CNS efficacy. Case reports focused on LMD have shown impressive responses with ALK targeting (59, 60), but larger studies are still needed.

In breast cancer patients with LMD the majority of targeted therapy efforts have been focused on HER2+ patients. HER2 is a tyrosine kinase in the EGFR family; its overexpression has a known impact on patient prognosis and is associated with CNS dissemination (61). Among available agents, the HER2 antibody trastuzumab has received the most attention, as pivotal trials demonstrated its efficacy in HER+ breast cancer (62). However, the BBB penetration of trastuzumab is limited, making the CNS a frequent site of progression (63). In light of this, two arms of investigation have evolved to address HER2+ CNS disease (including LMD). First, new HER2 TKIs with better BBB penetration, including lapatinib, neratinib and tucatinib, are currently been evaluated. For example, lapatinib is a small molecule that binds HER2; it has shown limited CNS efficacy as a single agent (64), but encouraging results have been observed when used in combination with capecitabine (65–68). The recent HERCLIMB Trial (291 HER2+ patients with brain metastasis enrolled) evaluated the intracranial efficacy of tucatinib when combined with trastuzumab and capecitabine. This trial demonstrated that the combination of tucatinib, trastuzumab, and capecitabine significantly reduced the risk of intracranial progression or death (HR 0.32; p<0.001) and increased the intracranial response rate compared to control group (47 versus 20.0%; p = 0.03*)*. However, this trial did not include LMD patients (69). For LMD specifically, Pellerino et al. reported the outcome of patients with HER2+ breast cancer LMD treated with neratinib. This study reported an OS of eight months, neurological improvement in 27% of cases, and stabilization of radiographic disease in 57% of cases (70). Second, to overcome the issue with BBB penetration, IT administration of HER2 targeted therapy has been explored in patients with HER2+ breast LMD. Of the available agents, IT administration of trastuzumab has been the most heavily evaluated. In a retrospective study by Figura et al., the authors compared the therapeutic efficacy of IT trastuzumab to IT methotrexate or whole brain radiation alone. IT trastuzumab resulted in significantly longer progression free survival and OS compared with the other treatment groups. Notably, 44% of patients treated with IT trastuzumab were alive at 6 months (71). A recent meta-analysis of 58 patients treated with IT trastuzumab reported radiological improvement in over 70% of patients and an OS of 13.2 months (72). Overall, IT trastuzumab is a potentially safe effective treatment of HER2+ LMD, but larger prospective studies needed.

Triple negative breast cancer (TNBC) presents a unique challenge as patients with this tumor type have limited targeted therapy options and a high risk of CNS dissemination. Poly adenosine diphosphate ribose polymerase enzymes (PARP enzymes) naturally repair the DNA breaks/damage that lead to apoptosis of tumor cells. BRCA (breast cancer susceptibility genes 1 or 2; BRCA) mutations impair the ability of PARP enzymes to repair damaged DNA. PARP inhibitors such as iniparib, olaparib, talazoparib and veliparib work by preventing tumor cells from repairing, allowing them to proceed to cell death. These inhibitors have been evaluated in patients with TNBC including those with brain metastasis. The use of PARP inhibitors has not yet been evaluated on a large scale in LMD, however, limited case reports have shown good clinical and radiographic response with olaparib (73, 74).

Finally, in melanoma, approximately 40-50% of patients with melanoma harbor a mutation in the serine/threonine kinase BRAF (75). As such, therapies targeting BRAF and MEK has significantly impacted the natural history and outcome of patients with BRAF mutant melanoma, even in those with CNS metastases. However, for patients with LMD, no prospective clinical trial using BRAF/MEK inhibitors has been performed to date, and data is limited mainly to case reports. Some of these outcomes have been very encouraging, as patients had prolonged survival; however, all these patients were BRAF/MEK treatment naïve when they were diagnosed with LMD and started on targeted therapy. This is important when assessing the available data, as patients who develop LMD while on targeted therapy continue to face a dismal prognosis (70). In addition, recent analyses of two large cohorts of patients confirmed that despite the use of targeted therapy, overall survival remained poor (76, 77).

## Role of Immunotherapy in LMD

Recent advances in the field of immunotherapy, specifically the use of checkpoint inhibitors, has led to improved outcomes across multiple malignancies, including in patients with advanced disease. Importantly, checkpoint inhibitors have shown to provide improved outcomes in patients with brain metastases, specifically in patients with melanoma brain metastases (78–80). Until recently, patients with LMD have been excluded from trials for patients with brain metastases, and the limited data for systemic checkpoint inhibitor therapy was based on case reports only (81–83). The ABS trial focused primarily on the checkpoint inhibitor treatment (nivolumab) of melanoma patients with parenchymal brain metastases and also allowed patients with LMD to be enrolled into cohort C, which also included patients who had failed local therapy, were symptomatic and required steroids. None of the 4 patients with LMD obtained benefit from treatment with nivolumab (79). Two recent prospective trials were designed specifically for patients with cancer and LMD, and both of them used systemically administered pembrolizumab, another checkpoint inhibitor. The first one, a phase 2 trial, mainly enrolled patients with breast cancer (n=17), lung cancer (n=2) and ovarian cancer (n=1) (84). The primary endpoint was OS at 3 months, with secondary endpoints being toxicity and response. With a median follow-up of 6.3 months (range, 2.2-12.5 months) in surviving patients, the primary endpoint was met, and 12 out of 20 patients were alive 3 months after enrollment. No new safety signals were observed with the use of systemic pembrolizumab in this patient population. A second phase 2 study by Naidoo et al. enrolled 13 patients across multiple pathologies and reported a CNS response rate of 38% and a median OS of 4.9 months (85). Importantly, these patients were all treatment naïve to checkpoint inhibition with anti-PD1. TNBC, which has limited targeted therapy options, has ongoing trials evaluating the therapeutic efficacy of checkpoint inhibition, specifically PD-1 and PDL-1 inhibition, in combination with stereotactic radiosurgery (NCT03807765, NCT03449238, NCT03483012). In addition, another trial is evaluating the safety and dose limiting toxicity of combining avelumab in combination with whole brain radiation for patients with LMD and any type of solid tumor history (NCT03719768).

The effect of the combination of ipilimumab and nivolumab on survival has also been assessed in a phase II trial 18 patients with LMD (86). These patients [with breast cancer (n=8), melanoma (n=2), lung (n=2) and other primary tumors (n=6)] were treated with the combination of ipilimumab and nivolumab. Importantly, the majority of patients (78%) required corticosteroids for symptom management at treatment initiation or any point during the trial. Like the prior trial with single agent anti-PD1, this trial met its primary 3-month OS endpoint, as 8 patients (44%) were alive at that time point. Median survival for the whole group of patients was 2.9 months (90% CI: 1.6- 5 months). Six patients (33%) had grade 3 or 4 treatment related toxicities, which is slightly less than previous reports, but importantly, no new safety signals were seen.

In addition to systemic administration, IT approaches using various immunotherapies have been investigated for the treatment of LMD. In a retrospective review of 178 melanoma patients, the authors reported that patients treated with IT therapy were afforded significantly longer survival time (76). Interestingly, the IT use of immunotherapy dates back to the 1980s, when IT interleukin 2 (IL-2) was first assessed in patients with melanoma LMD. Two large cohorts (n=46 n=43) of patients with melanoma reported a median OS of 11.5 months (range 7-19), as well as 1-year, 2-year and 5-year OS rates of 36%, 26% and 13%, respectively, in patients that responded to IT IL-2 (87, 88). However, this approach is associated with significant toxicities the need for prolonged and intensive in-hospital monitoring, making this therapy an option for only a highly selected group of patients. To avoid the toxicity associated with IT IL-2, and based on the fact that systemic anti-PD1 administration leads to higher response rates than high dose IL-2 in patients with metastatic melanoma, a recent first-in-human trial assessed the safety and initial efficacy of using IT nivolumab in combination with IV nivolumab (NCT03025256). This allowed for a dose intensified approach, as it was shown that systemically administered nivolumab has very poor CSF penetration (89). Initial results for the first 25 patients treated showed a median OS of 4.9 months at a median follow-up of 20 weeks (range 5-147), with 3, 6 and 12 months OS rates of 65%, 47% and 35%, respectively. Importantly, the use of IT nivolumab was very well tolerated with no grade 3 or 4 toxicities observed, and the addition of IV nivolumab did not increase the expected toxicity from systemic anti-PD-1 administration. The trial is ongoing, with translational data expected in the near future (90).

## Conclusion

LMD represents a subtype of CNS metastatic disease with few treatment options, and patients diagnosed with LMD, as well as their treating physicians, face major challenge in both prompt diagnosis and treatment. This condition commonly results in rapid neurologic disability and death. Even with currently available therapies, prognosis remains grim. However, multiple avenues, including targeted and immunotherapy, are currently being explored to combat this challenging disease and gain a deeper insight into the underlying disease pathogenesis. Much more needs to be learned and done, but the initial results of these new approaches are promising.

## Author Contributions

SF: Conception, drafting of manuscript and review of final version. EF: Drafting of manuscript and review of final version. RG: Review of final version. IG: Conception, drafting of manuscript and review of final version. All authors contributed to the article and approved the submitted version.

## Conflict of Interest

The authors declare that the research was conducted in the absence of any commercial or financial relationships that could be construed as a potential conflict of interest.

## Publisher’s Note

All claims expressed in this article are solely those of the authors and do not necessarily represent those of their affiliated organizations, or those of the publisher, the editors and the reviewers. Any product that may be evaluated in this article, or claim that may be made by its manufacturer, is not guaranteed or endorsed by the publisher.
